# The Relation of Spirochæta Pallida to Syphilis

**Published:** 1906-02-03

**Authors:** 


### THE RELATION OF SPIROCHÆTA PALLIDA TO SYPHILIS.

Speaking at the Medico-Chirurgical Society of Edinburgh, Dr. Theodore Shennan stated that while recent observations pointed to an etiological relationship between S. pallida and syphilis, some reserve must be expressed in accepting the descriptions of some of the observers. The true Spirochæta pallida was rarely present in large numbers in one preparation and all material derived from superficial sores was liable to contamination with the much more easily detected S. refringens. In their typical forms these organisms can be easily distinguished from one another, but in many preparations a number of intermediate forms are present which are difficult to assign to one or other species, especially when few in number. The method of Giemsa is at present the only differential stain, and in films treated in this way S. pallida is stated to take a rose-pink while S. refringens stains a purple colour. This does not form a sufficient distinction, as varying depths of staining as well as varieties in form may be met with in the same preparation. In procuring films from surface lesions it is important to cleanse the surface very thoroughly so as to avoid S. refringens and obtain more secretion from the deeper layers in which S. pallida is more abundant. Considerable scepticism should be exercised as to the numerous observations of S. pallida where it is stated that only one or two organisms were found, even when they stain pink with Giemsa's solution. A reliable differential stain is much to be desired, but in the meantime only the most conclusive observations justify one in asserting the presence of true Spirochæta pallida in any lesion.

